# Notes from Our Scrap-Book

**Published:** 1882-01

**Authors:** 


### NOTES FROM OUR SCRAP-BOOK.

It may be well to explain how the writer came to compile these notes and their use to him.

Early in my study of the materia medica, a veteran in the service told me the best method for getting a critical knowledge of drugs, was to study the symptoms which many drugs possessed in common, and to note their differences. He said, "Much of this will be forgotten, but what is remembered will be of great value." This plan has been followed and found very useful; instead of merely reading and comparing these symptoms, they have been written down for future reference. Having been of service to the compiler, and to a few who have seen them, it is hoped others may find them useful.

Some few remedies are here given, which have *bearing down pains, pressure,* etc., in abdomen, in connection with menstrual disorders, taken chiefly from Hering, Jahr and Lippe.

*Actea rac.*—Bearing down pain in uterine region and small of back; limbs heavy and torpid.

*Agaricus*—Bearing down pain is intolerable.

*Aloe*—Pressing down in rectum.

*Ant. crud.*—Pressure in uterus as if something would come out.

*Apis*—Great tenderness over uterine region, with bearing down pain; micturition painful.

*Arg. nit.*—Pressure in præcordial region, at night during menses.

*Asafœt.*—Bearing down in genitals, worse riding in carriage; labor-like pains during menses.

*Bellad.*—Pressure downwards as if contents of abdomen would come out from the vulva (*Sepia*); worse in early morning, and when sitting *bent* forward, or walking; better standing or sitting *erect.*

*Bovista*—Bearing down in vagina and weight in small of back, after midnight.

*Calc. phos.*—Throbbing, stinging, tickling, sore aching, or pressing, in the genitals, drawing upwards to the symphysis, downwards into the thighs.

*Cantharis*—Violent pinching pains with bearing down to genitals; great burning pain.

*Carbo an.*—Labor-like pain in sacrum and pelvis; pressing in small of back, groins and thighs; chilly, yawning and desire to urinate.

*Cauloph.*—Weight, heaviness in uterine region.

*Cham.*—Drawing from small of back forwards, griping and pinching in uterus followed by a discharge; labor-like pains during menses.

*China*—Pressing down, worse when walking.

*Cocculus*—Painful pressing in uterine region, with cramps in chest, fainting, nausea, and weakness.

*Colocynth.*—Bearing down like cramps, which cause her to bend double (*Opium*).

*Conium*—Pressure from above down, drawing in legs during menses.

*Cuprum*—Before or during menses, or after suppression of menses (from suppressed foot-sweats, also *Silicea*), violent cramps in abdomen and *into chest.*

*Digitalis*—Labor-like pains in abdomen and back before menses.

*Drosera*—Labor-like pain in abdomen.

*Ferrum*—Sharp pains in abdomen; bearing down in uterine region, with aching.

*Gelsem.*—Bearing down in abdomen, uterus feels as if *squeezed by a hand*; heaviness in uterine region; pain from back into thighs.

*Graphites*—Bearing down pain in abdomen to back, with weakness, painful pressure to pudenda; labor-like pains.

*Helonias*—Dragging weakness in sacral region (prolapsus) with melancholy humor; soreness and weight in womb; "consciousness of a womb."

*Hepar S. C.*—Weight, congestion in womb.

*Hyoscyamus*—Uterine cramps, pulling in loins and small of back.

*Hypericum*—Tension as from bandage around abdomen; pressure in small of back and lower bowels.

*Iodium*—Dull, pressing, wedge-like pains from right ovary to uterus.

*Kali bichr.*—Dull, pressing, heavy pain in hypogastrium, pain and weakness in small of back.

*Kali carb.*—Pain like a weight in small of back.

*Kreasotum*—Bearing down and weight in pelvis; painful urging to the genitals.

*Lachesis*—Cannot bear even weight of sheet on genitals, so sensitive (*Lilium, Mur. ac., Puls.*); bearing down pains; labor-like pains.

*Lilium*—Bearing down when standing; labor-like pain in left ovarian region and left mammæ; cannot bear any weight on genitals; pain in sacrum.

*Lobelia*—Sense of weight in genitals; pain in sacrum.

*Lycop.*—Sense of pressure through vagina when stooping.

*Mercurius*—Abdomen weak as if it had to be held up; dragging in loins.

*Natr. c.*—Pressure in hypogastrium as if everything would come out.

*Natr. m.*—Every morning pressure and pushing towards genitals; must sit down to prevent procidentia (must cross legs, *Sepia*).

*Nitr. ac.*—Pressing down in hypogastrium and small of back as though everything would protrude.

*Nux mos.*—Bearing down pain in abdomen, drawing in limbs; pain from small of back downwards; pain in small of back as from a piece of wood pressed out; mouth always dry.

*Nux vom.*—Pressure to genitals in morning; bearing down to sacrum with urging to stool.

*Platina*—Much bearing down; painful sensitiveness; continued pressure in uterine region.

*Plumbum*—Bearing down pains; sensation as of a string pulling abdomen towards back.

*Podophyl.*—Sensation as if genitals would come out, during stool; menses suppressed with bearing down in hypogastric and sacral regions, worse from motion.

*Pulsat.*—Pressure in abdomen and small of back as from a stone.

*Rheum*—Bearing down in uterine region while standing (*Lilium*).

*Rhus tox.*—Bearing down when standing or walking; backache, better lying on something hard.

*Sabina*—Labor-like pain from sacrum to pubis, colic.

*Sepia*—Pressing in uterus, causing oppression of breathing (*Pulsat.?*), from above downward, with colicky pains; feels if everything would come out (*Bellad.*, *Natr. c.*, *Natr. m.*, *Nitr. ac.*)

*Silicea*—A pressing down feeling in vagina.

*Sulphur*—Bearing down in pelvis towards genitals.

*Ustilago*—Pressure as if everything would come out. E. J. L.

